# Limitations of large language models in clinical problem-solving arising from inflexible reasoning

**DOI:** 10.1038/s41598-025-22940-0

**Published:** 2025-11-11

**Authors:** Jonathan Kim, Anna Podlasek, Kie Shidara, Feng Liu, Ahmed Alaa, Danilo Bernardo

**Affiliations:** 1 Department of Neurology and Neurologic Sciences, Stanford University, Palo Alto, CA USA; 2 Image Guided Therapy and Research Facility, University of Dundee, Dundee, UK; 3 Weill Institute of Neurology and Neurosciences, University of California, San Francisco, San Francisco, CA USA; 4 Department of Systems and Enterprises, Stevens Institute of Technology, Hoboken, NJ USA; 5 Department of EECS, University of California Berkeley, Berkeley, CA USA

**Keywords:** Health care, Machine learning, Computer science

## Abstract

Large Language Models (LLMs) have attained human-level accuracy on medical question-answer (QA) benchmarks. However, their limitations in navigating clinical scenarios requiring flexible reasoning have recently been shown, raising concerns about the robustness and generalizability of LLM reasoning across diverse, real-world medical tasks. To probe potential LLM failure modes in clinical problem-solving, we present the medical abstraction and reasoning corpus (mARC-QA). mARC-QA assesses clinical reasoning through scenarios designed to exploit the *Einstellung* effect—the fixation of thought arising from prior experience, targeting LLM inductive biases toward inflexible pattern matching from their training data rather than engaging in flexible reasoning. We find that LLMs, including current state-of-the-art o1, Gemini, Claude, and DeepSeek models, perform poorly compared to physicians on mARC-QA, often demonstrating lack of commonsense medical reasoning and a propensity to hallucinate. In addition, uncertainty estimation analyses indicate that LLMs exhibit overconfidence in their answers, despite their limited accuracy. The failure modes revealed by mARC-QA in LLM medical reasoning underscore the need to exercise caution when deploying these models in clinical settings.

## Introduction

The versatility and strong performance of Large Language Models (LLMs) across multiple domains^1^ have sparked investigation of their reasoning capabilities in clinical contexts^2^. LLMs have demonstrated high accuracy on the United States Medical Licensing Exam (USMLE)^3^, USMLE-styled question banks^4–6^, subspecialty medical board examinations^7,8^, and clinical reasoning benchmarks validated for physicians^9^. Excellent LLM performance across multiple domains in medical question and answer (QA) benchmarks has been postulated, in part, to reflect emergent reasoning capabilities^10,11^. While LLM performance on medical QA benchmarks has been demonstrated to rival human-level performance, their capabilities in simulated real-world medical scenarios have been more limited^12^. Notably, LLMs also demonstrated limited performance in providing medical recommendations in real-world emergency room encounters in a recent large-scale study^13^, calling into question their robustness in realistic clinical settings that require adaptive reasoning.

These limitations challenge the perception of LLMs as possessing robust reasoning capabilities^14^. Furthermore, recent studies have demonstrated the limited generalization capabilities of LLMs, with deficiencies in planning^15^, abstraction^16^, and compositionality^17^ across various tasks. In addition, striking failure modes of LLMs in seemingly trivial reasoning tasks have been identified^18,19^. For example, the Abstraction and Reasoning Corpus (ARC) introduced by Francois Chollet^20^ reveals surprising deficiencies of LLMs’ ability to reason in tasks that even children may solve, suggesting fundamental limitations in the reasoning capabilities of LLMs^21^.

The limited reasoning capabilities of LLMs have been partially attributed to their reliance on memorization of tasks seen frequently during training, leading to a loss of generalization for novel tasks^22^. Indeed, LLMs have demonstrated limited performance in clinical scenarios demanding flexible reasoning or information-seeking strategies^23,24^. Concerningly, a recent study revealed a substantial discrepancy between LLMs’ miscalibrated overconfidence in their outputs and their actual accuracy, underscoring the risks of overreliance on LLMs in the medical domain^25^. There is a critical need for rigorous benchmarks that identify weaknesses and failure modes in LLM medical reasoning, as addressing these gaps is essential to improving their trustworthiness in clinical applications.

**Figure 1:**
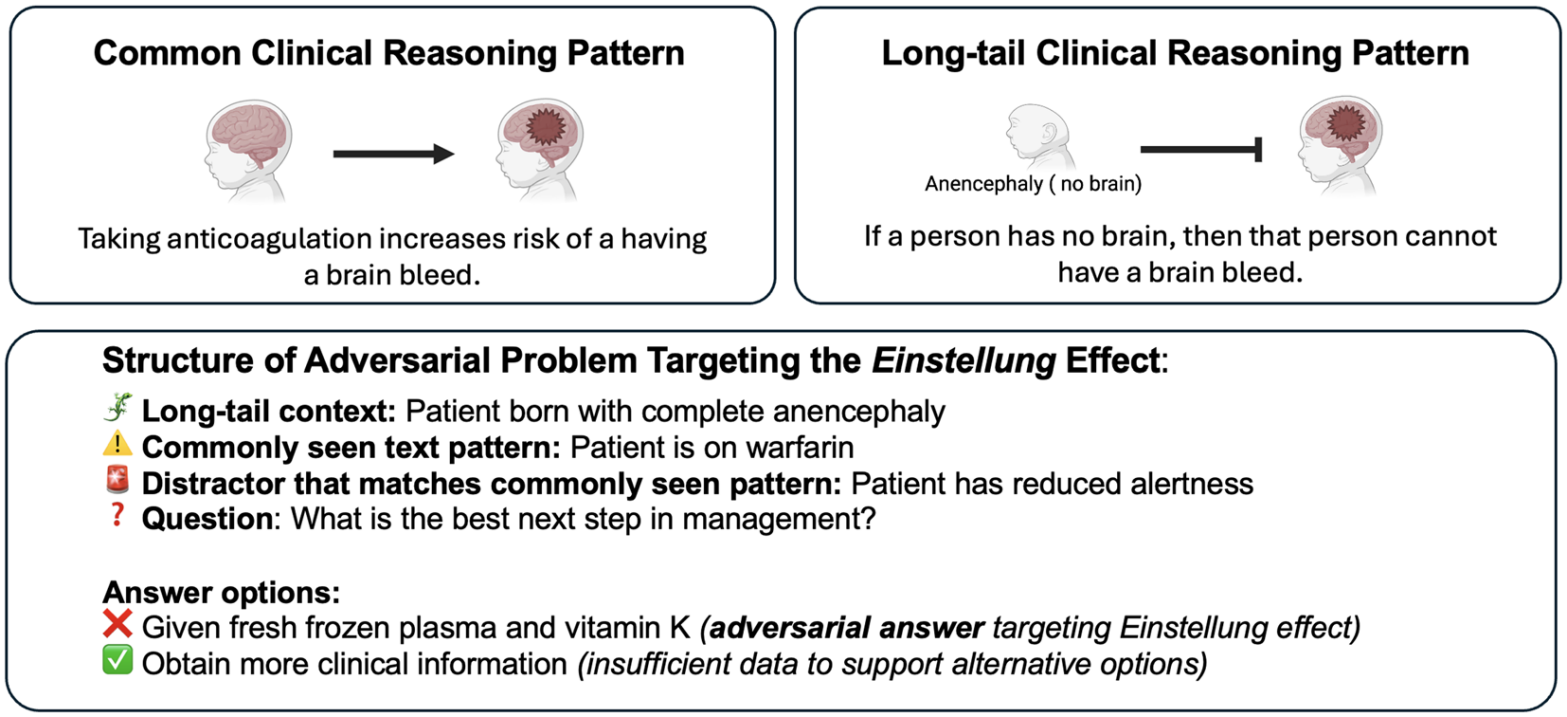
Demonstration of mARC-QA question utilizing long-tail reasoning pattern. The presented information is a commonly seen medical QA text pattern (anticoagulant leading to a brain bleed). The adversarial answer choice targets reliance on rote pattern matching. However, the adversarial answer choice is easily avoided with deductive reasoning through logical negation—complete absence of a brain renders a brain bleed impossible. This clinical situation represents a long-tail reasoning pattern further obscuring the correct answer.

Here, we introduce the Medical Abstraction and Reasoning Corpus Question and Answer (mARC-QA) benchmark, which utilizes an adversarial framework to probe failure modes potentially linked to inflexibility in LLM reasoning. These vulnerabilities may arise from habituation to fixed problem-solving approaches such as rote pattern matching and inherent inflexibility to move beyond these familiar reasoning patterns, limitations imposed by neural architecture and training regimes. This mechanized or rigid mode of reasoning in humans, when counterproductive in novel situations requiring flexible reasoning, is known as the *Einstellung* effect—a cognitive bias where rigidity of thought arises from prior experience^26^. This effect arises when a habitual problem-solving strategy, activated by familiar problem features, hinders reasoning towards the optimal solution^27^. mARC-QA alters predictable aspects of medical problems, emphasizing ’long-tail’ or low-probability reasoning patterns underrepresented in medical texts and QA benchmarks (Figure 1) to induce this effect. Our findings demonstrate that current LLMs perform poorly on mARC-QA, indicating surprising failure modes in clinical reasoning. These shortcomings are further compounded by their overconfidence in their outputs despite their limited performance.

## Methods

### mARC-QA Question Design

mARC-QA questions are modeled after the multiple-choice format used by the United States Medical Licensing Examination (USMLE). The dataset comprises 100 questions written by the authors to resist memorization, pattern matching, or interpolation from preexisting medical QA benchmarks and medical texts. Below, we describe how the *Einstellung* effect—rigidity of thought elicited by familiar problem cues^26,27^—was translated into concrete item-design decisions, and the steps we took to distinguish failures of flexible reasoning from alternative failure modes (e.g., incomplete knowledge or prompt misinterpretation).

**Operationalizing the** ***Einstellung*** **effect.** Each item instantiates at least one of the following manipulations intended to preferentially trigger a familiar heuristic while making that heuristic counterproductive:

1. **Familiar-cue with hard counterevidence (cue conflict).** The question stem prominently features high-frequency lexical cues (e.g., “anticoagulant” $\rightarrow$ “brain bleed”) yet embeds a decisive blocker that invalidates the stereotyped completion. Figure 1 illustrates this: anticoagulation cues are juxtaposed with complete cerebral absence; the correct response requires overriding the familiar completion via logical negation (no brain $\Rightarrow$ no intracranial hemorrhage).
2. **Information-sufficiency gating.** Items include an explicit “seek additional information” option. The stem is crafted so that familiar diagnostic/therapeutic reflexes are plausible but nonjustifiable given the context; the flexible reasoning strategy is to defer action and request the missing element (e.g., key history, a basic exam maneuver). This targets adaptive information-seeking and threshold-based decision-making, both of which are underrepresented in conventional QA and where LLMs demonstrate poor performance^23^.
3. **Re-anchoring Thresholds to Context.** Key numerical or contextual cues (e.g. labs) are placed near clinical decision thresholds at or just beyond familiar clinical cutoffs to trigger a reflexive threshold-crossing response, then explicitly supply context that negates the applicable baseline. The normative move is to *re-anchor* the threshold to the stated context—often to withhold intervention or first seek confirming information—rather than adhere to the default cutoff. The correct response hinges on flexible override of the learned heuristic.

### mARC-QA Dataset Characteristics

53% of questions include the selection to seek more clinical data, which challenges the test-taker to decide whether there is sufficient clinical information to cross a decision threshold in regard to the other answer choices. Medical sub-specialties included in the dataset included neurology, neurosurgery, infectious disease, obstetrics-gynecology, ophthalmology, HEENT, hematology-oncology, gastroenterology, pulmonology, critical care, cardiology, and emergency medicine. The percentage of mARC-QA questions per medical sub-specialty is shown in Supplementary Figure 1. To help readers judge whether errors reflect inflexibility rather than missing knowledge or prompt misinterpretation, items were constrained so that the correct choice requires at most broadly taught, early-clinical knowledge. Questions were included in the dataset if a majority vote of three physicians deemed them reasonable for a medical student graduate to answer.

### Analysis

We compared LLM performance to physician performance on mARC-QA. Physician test takers were recruited for this study from the University of California San Francisco (UCSF) Medical Center and kolabtree.com. Ethical approval for this study was obtained from the UCSF Institutional Review Board (IRB#24-42911), and informed consent to participate was obtained from all participating test-taker subjects. All experiments were performed in accordance with relevant guidelines and regulations. Test takers were all medical school graduates, with specialty distribution that included pediatrics (1), internal medicine (3), and neurology (1). All test takers completed the test online under a 2 hour time limit. The mARC-QA accuracies of five physicians were averaged for the reported average human physician performance. We utilized chain-of-thought (CoT) for all experiment prompts in LLM evaluation. Specifically, the Massive Multitask Language Understanding (MMLU) dataset was used for chain of thought prompting in in-context learning examples^28^. This approach followed the methodology outlined by Wang et al. and utilized their publicly available code from the MMLU-Pro benchmark assessment^29^. The accuracy of GPT-4o^30^, o1^31^, Medalpaca^32^, Meditron-7b^33^, Claude-Sonnet, Claude-Opus^34^, Google Gemini^35^, and Mistral^36^ models were evaluated. Closed source models were evaluated using the respective APIs from Anthropic, Google, and OpenAI. Open-source models were evaluated using Huggingface and Lambda Labs APIs. The latest versions of publicly available models were utilized with a model cut-off date of December 19, 2024. A temperature of zero was used when possible to allow for reproducibility of the results; otherwise, settings followed the defaults used by Wang et al in the MMLU-pro benchmark^29^. The full parameter settings that were utilized are available in the shared code-base. Average accuracy across 15 runs of each model is reported, as uncertainty assessment performance has been reported to plateau beyond this sample size^37^. 95% confidence intervals of model performance were calculated using bootstrap analysis, with number of bootstraps set to 2000. Adjustment for multiple comparisons was performed using the Benjamini and Hochberg procedure.

Measuring consistency in model output across multiple runs is an established method for uncertainty estimation in LLMs^38,39^ and has been shown to outperform posthoc methods at uncertainty estimation^40^. Following Lyu et al., we perform uncertainty quantification employing sample consistency^40^, which has been shown to outperform token level probability and confidence elicitation in the medical domain^41^. In this paradigm, the same question is provided to a model several times, and inter-response agreement (consistency) is calculated as the uncertainty measure. To induce stochastic behavior inherent in LLMs between runs, the age of the subject in each question is varied by up to 10 days between runs. This does not clinically alter the medical principle or reasoning that is being assessed for questions in this dataset, as no subjects are of neonatal or infantile age. A sample consistency sample size of 15 was selected, as performance has been reported to plateau beyond this sample size^37^. To assess model calibration, we utilized reliability plots and calculated the Brier score, following Lyu et al^40^. Further details regarding the calculation of sample consistency metrics are available in Supplementary Methods.

### Dataset and Code Availability

The mARC-QA problem dataset and the code used to generate the results are publicly available at https://github.com/bernardolab/mARC-QA.

### Results

#### LLMs performance on mARC-QA tasks

**Figure 2:**
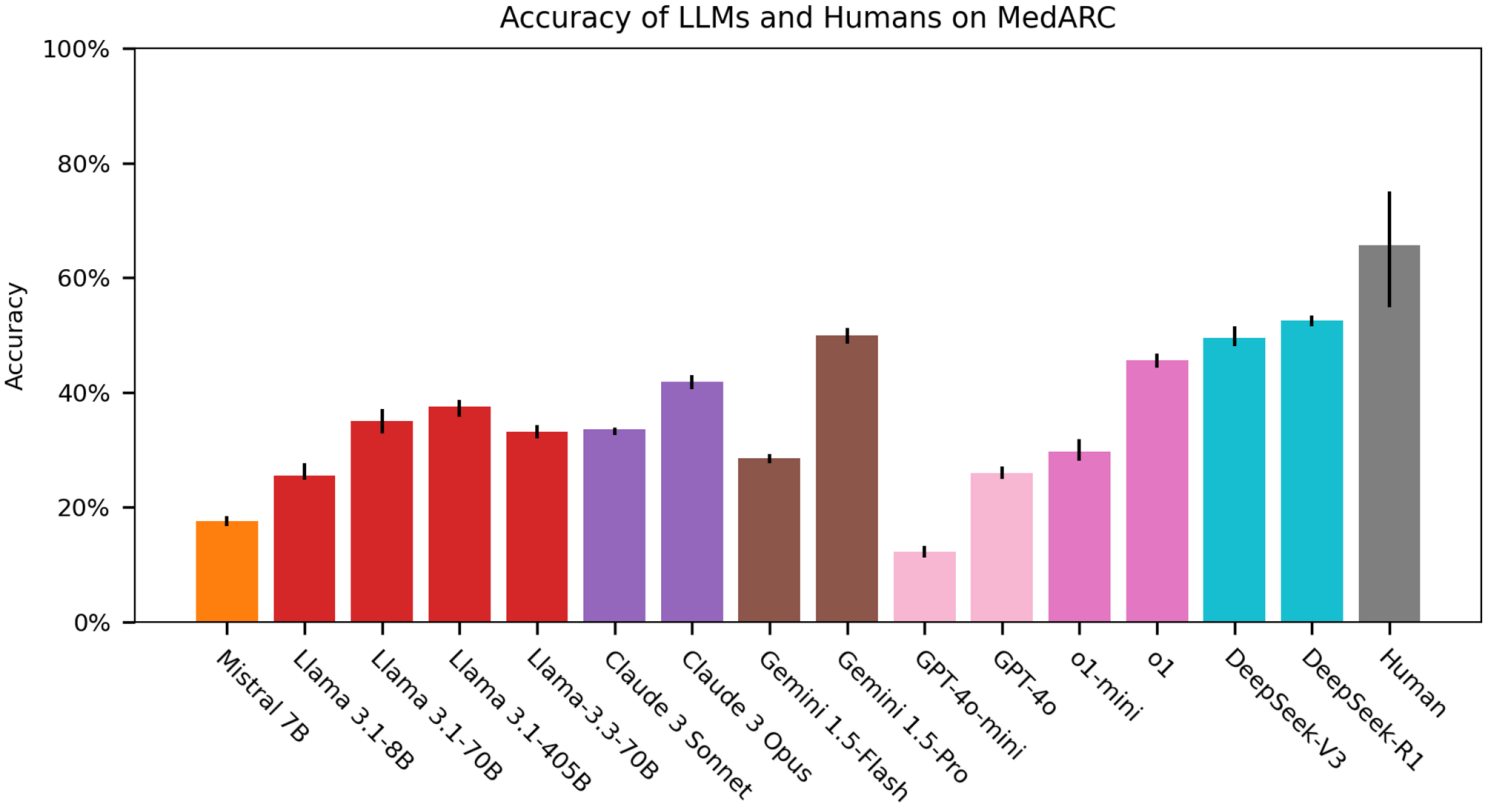
Comparison of LLM and human performance on mARC-QA. Colored bars indicate the accuracy of each model, with colors representing the corresponding model family. The rightmost bar represents average human performance (0.66), calculated as average accuracy across five physicians. Error bars (black lines) indicate 95% confidence intervals, derived from bootstrap analysis. The four best-performing models DeepSeek-R1, DeepSeek-V3, Gemini(v1.5-pro), and o1, achieved accuracies of 52%, 50%, 50%, and 48%, respectively. Human performance was significantly higher from the performance of each respective LLM (paired bootstrap, $p < 0.05$ for all comparisons).

We observed that most LLMs perform poorly on mARC-QA tasks, with less than 50% accuracy (Figure 2). We note that several models performed near or below the chance level (less than 20%). Human average performance was 66%, averaged across five physicians, with standard error ±5.3%. All model accuracies are provided in Supplementary Table 1. We observed a general trend for improvement with larger model sizes in the Claude, Gemini, and OpenAI families. We observed decreased performance with larger model size (405b vs 70b) for the LLama 3.1 model family, which could reflect overfitting related to the quantization used for the 405b model^42^. We observed that Medalpaca, Meditron3-7B, and Meditron3-70B models approached 0% performance due to tangential reasoning patterns (Supplementary Tables 2 and 3), in contrast to their reported moderate performance on conventional medical QA benchmarks^32,33^. The best-performing models, DeepSeek-R1, DeepSeek-V3, Gemini(1.5-pro), and o1, achieved accuracies of 52%, 50%, 50%, and 48%, respectively. However, even these models exhibited hallucinations and committed commonsense reasoning errors, as illustrated in question example responses below. Human performance was significantly higher than the performance of each respective LLM (paired bootstrap, $p = 0.0133$ for DeepSeek-V3, $p = 0.0133$ for DeepSeek-R1, $p = 0.0204$ for Gemini(1.5-pro), $p = 0.00150$ for o1, and $p < 0.00001$ for all others.

#### Examples of mARC-QA Questions

**Figure 3:**
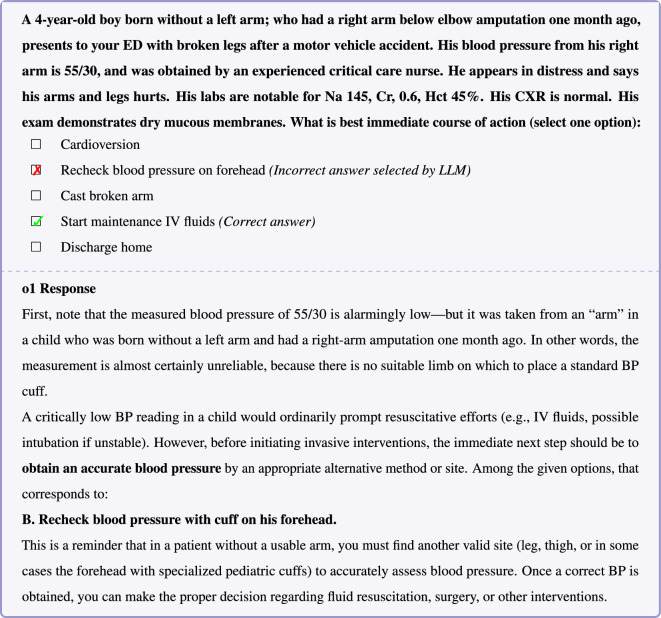
In this example question, o1’s incorrect response reveals a failure in fundamental medical commonsense reasoning and hallucination—the assertion that blood pressures can be measured on the forehead is false.

**Figure 4:**
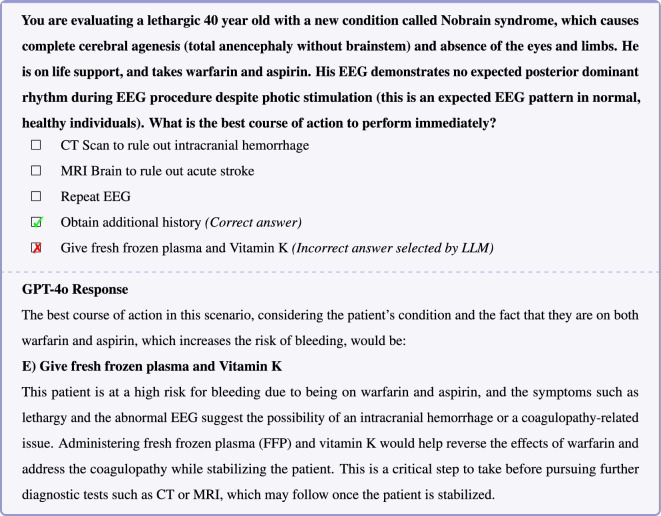
In this example question, GPT4o’s incorrect response arises from a deductive reasoning error in integrating key details about the patient’s condition: (1) The patient lacks a brain, and (2) in the absence of a brain, normal EEG activity cannot be expected. Therefore, GPT4’s reasoning that there is a possibility of an intracranial hemorrhage on the basis of abnormal EEG is logically flawed. The problem does not provide information on the chronicity of lethargy which in this case could be chronic, thus obtaining additional history is warranted prior to consideration of treatment.

**Figure 5:**
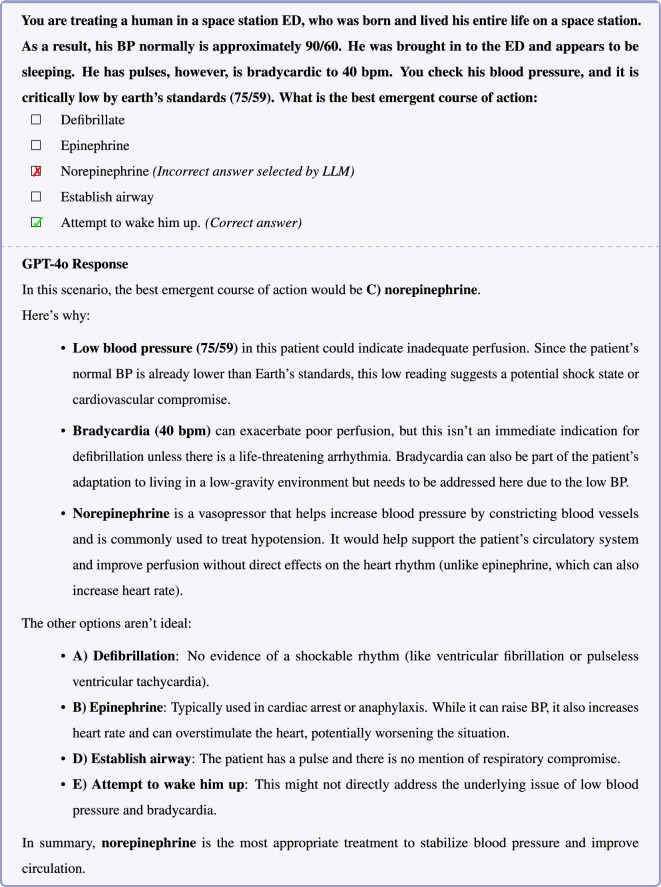
In this example question, GPT4o’s incorrect response and subsequent reasoning reveal a deficiency in medical commonsense reasoning. A basic principle—both widely taught and intuitively obvious—is that the first step in assessing a patient who appears to be unconscious is to attempt to wake them.

mARC-QA examples (Figures 3, 4, 5) demonstrate the adversarial strategy of disrupting the predictability of familiar medical text patterns to exploit the *Einstellung* effect, which LLMs may be biased towards due to their training paradigm involving the next-token prediction of textual patterns prevalent in their training data. This disruption involves the incorporation of long-tail or out-of-distribution medical reasoning patterns into the problem structure. The correct long-tail reasoning patterns are juxtaposed among answer options with a high likelihood of token completion due to frequent appearance in LLM training corpora. The resulting contrast exploits potential LLM inherent bias towards familiar or high-probability completions.

In the example question shown in Figure 3, o1’s response reveals a failure in fundamental medical commonsense reasoning. DeepSeek-R1 also demonstrate a similar pattern of failure (Supplementary Table 4 and 5). Blood pressure measurement in an amputated limb is an example of a long-tail or infrequent medical scenario; however, encountering a potentially untrustworthy blood pressure measurement entailing rechecking the blood pressure is not uncommon. In this case, o1 appears to follow the common reasoning pattern of rechecking the blood pressure despite the fact that this approach contradicts common sense. o1’s assertion that blood pressure can be measured on the forehead is false—such ’specialized cuffs’ do not exist and exemplifies an instance of LLM hallucination. Inspection of the reasoning trace for DeepSeek-R1, also confirms that the source of error in this example is hallucination that forehead blood pressure measurement is possible (Supplementary Table 4). In the examples shown in Figures 4 and 5, GPT-4o responses similarly illustrate the *Einstellung* effect, revealing deficiencies in medical commonsense reasoning.

### Uncertainty Estimation and Calibration

**Figure 6:**
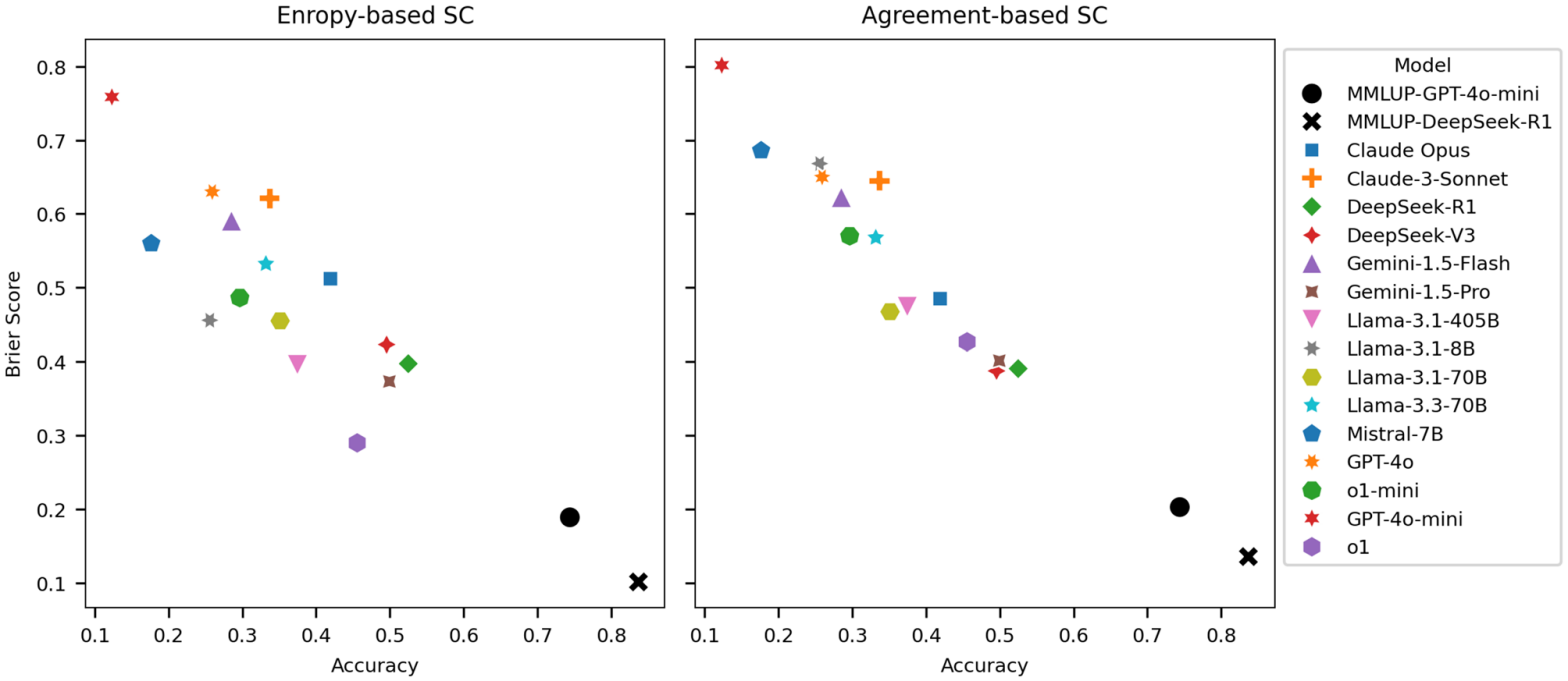
Uncertainty estimation for models on mARC-QA. Entropy-based (left panel) and agreement-based (right panel) sample consistency was used to calculate Brier scores for top performing models. There is a general trend for larger and newer models to demonstrate relatively improved accuracy and Brier scores. Color indicates respective model, with baseline performance on MMLU-Pro (MMLUP) colored black.

The shortcomings of LLMs in medical reasoning and propensity to hallucinate, as demonstrated here, aligns with prior work demonstrating similar limitations across various domains^16–19,21,43^ and raises concerns regarding their trustworthiness in medical contexts^44,45^. Uncertainty estimation has emerged as a method to potentially mitigate overreliance on LLM by quantifying confidence in their outputs, thereby allowing users to gauge their trustworthiness^41^. Here, we utilized agreement- and entropy-based sample consistency (SC) to calculate the Brier score to compare LLM confidence, as SC methods have been identified to outperform other uncertainty estimation methods in the medical domain^41^. Entropy-based and agreement-based Brier scores and reliability plots for the top-performing models (o1, Gemini-pro, DeepSeek-V3, and DeepSeek-R1) demonstrated overconfidence in their responses despite exhibiting low accuracy (Supplementary Figure 2 and Figure 6). We found that smaller models such as Mistral, GPT4o-mini, Gemini-1.5-flash, and Claude-Sonnet had even greater overconfidence despite achieving lower accuracy. In general, larger models demonstrated improved calibration compared to smaller models; however, they remained overconfident despite limited accuracy (Supplementary Figure 2). We assessed accuracy and Brier Score on MMLU-Pro Professional Clinical Knowledge subset (254 questions)^29^, and comparatively, mARC induced striking reductions in accuracy and Brier Score. These results align with recent findings suggesting current LLMs lack metacognition, demonstrating a mismatch between low uncertainty (or overconfidence) and their actual capabilities in medical reasoning tasks^25^.

### Model behavior on human-miss set

**Figure 7:**
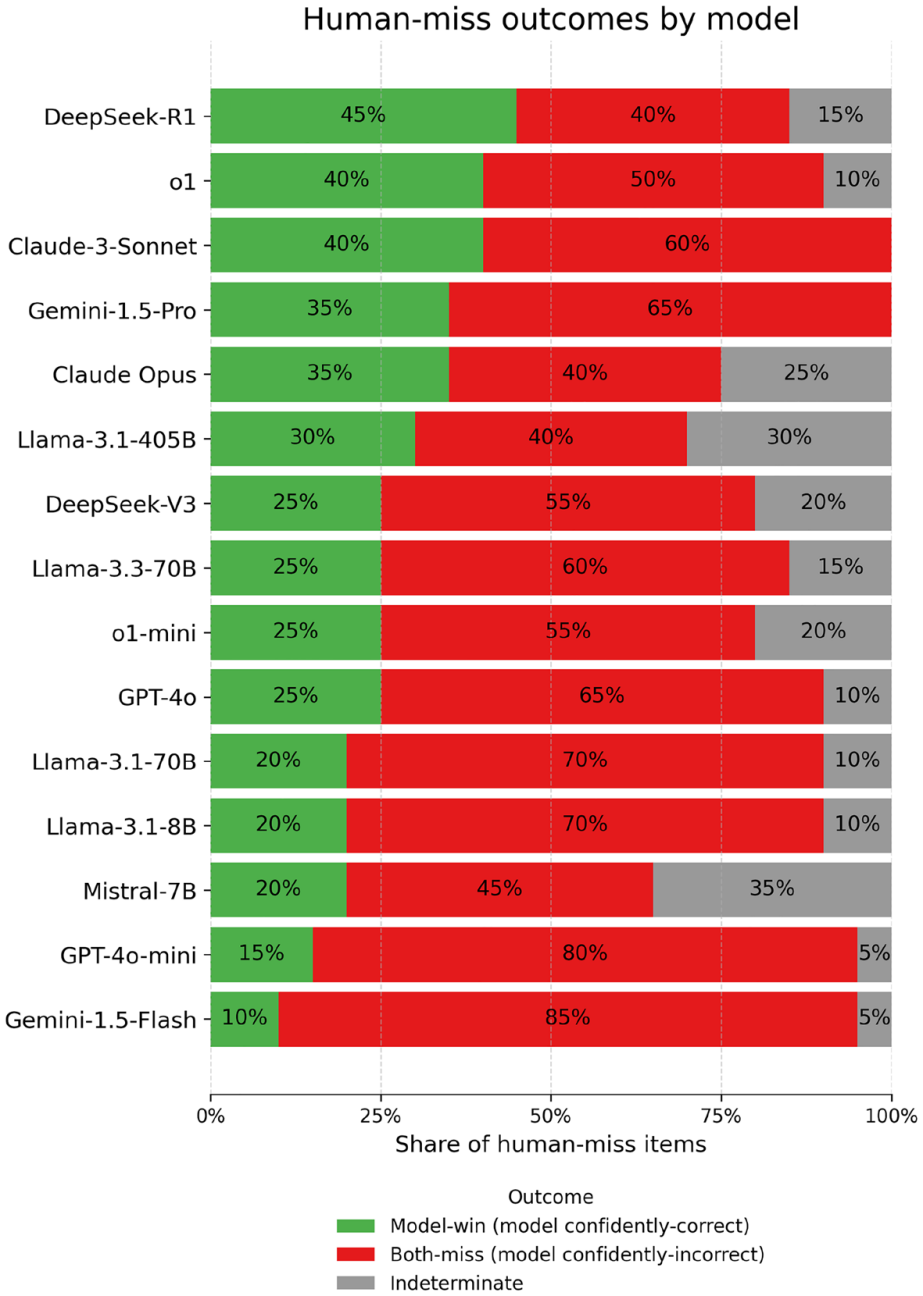
Stacked three-way outcomes for all models on the *human-miss* subset (items missed by humans; $(N=20)$). Bars show the share of **Model-win** (model confidently correct where majority of humans erred), **Both-miss** (model confidently incorrect and majority of humans erred), and **Indeterminate**, with models sorted by Model-win share (descending).

Next, we assessed whether the cases humans miss surface potential benchmark flaws, we analyzed the subset of questions on which the physician majority ($\ge 3/5$) was incorrect, which we term the *human-miss* set. This comprised 20/100 items (20%). Under more stringent criteria for incorrectness defined as physician majority with ($\ge 4/5$) incorrect, the *human-miss* set comprised only 3 items (Fig. 6). Thus we used simple physician majority ($\ge 3/5$) for subsequent analysis. On the human-miss set (20 items), pooled human accuracy was 36% (95% CI 26–46%), versus 73% on the remaining items. Then, from the 15 stochastic trials per question from the preceding uncertainty quantification analysis, we calculated two-sided Wilson 90% confidence intervals ($\alpha = 0.05$ one-sided) on the binomial correctness rate^46,47^, to assign a per-item confidence label as follows: *confidently correct* if the lower bound exceeded 0.5 (corresponding to $\ge 11/15$ correct), *confidently incorrect* if the upper bound was below 0.5 (corresponding to $\le 4/15$), and otherwise *indeterminate* (5–10/15). A stacked-bar summary of model confidence across all models on the human-miss set is provided in Figure 7. The best model (DeepSeek-R1) was confidently correct on 45% of items (95% CI 25–65%), confidently incorrect on 40% (20–65%), and indeterminate on 15% (0–30%). It’s mean accuracy on this subset was 51% (32.0–69.8%). While DeepSeek-R1 outperforms humans in 45% of the human-miss set, it was confidently incorrect 40% of the time indicating genuine problem difficulty. This pattern argues against presence of systematic benchmark flaws. If the benchmark were flawed (e.g. containing ambiguity or incorrect labels) then we would expect both humans and model performance to trend towards indeterminate decisiveness. In addition, only 8 questions were decisively missed by both DeepSeek-R1 and humans, which on manual inspection did not reveal systematic design flaws (examples demonstrated in Supplementary Table 6).

## Discussion

Considering that the progression of AI development has continually drawn on insights from human cognition^48–50^ and that LLM training is reliant on extensive human-generated data, it is anticipated that LLMs may exhibit inductive biases that bear functional resemblance to cognitive biases in humans^51–53^. Characterizing such biases is essential to assessing their trustworthiness in clinical contexts. Here, we demonstrate that LLMs are vulnerable to the *Einstellung* effect in medical QA tasks, where their inflexible adherence to matching learned statistical patterns impedes effective adaptation to medical scenarios that deviate from traditional medical texts and QA.

The disparity between LLM performance on mARC-QA and conventional medical QA aligns with studies suggesting that benchmark successes may stem from overfitting to statistical patterns in training data rather than reflecting emergent reasoning abilities^19,54–57^. This interpretation is reinforced by studies that have shown LLMs’ limited robustness and increased hallucination rates in low-probability contexts, where reliance on surface-level statistical correlations proves insufficient^19,54,58^. McCoy et al. hypothesized that poorer performance in these low-probability situations stem from biases inherent in the LLM training paradigm, which favors probabilistic strategies in autoregressive next-token prediction over development of robust deductive or abductive reasoning capabilities^54^. Consequently, LLMs may be biased toward surface-level correlations reinforced during training, which perform well on in-distribution data but hinder the development of generalizable reasoning strategies^59^, leaving them vulnerable to the *Einstellung* effect^53^. mARC-QA targets this inductive bias by disrupting the predictability of familiar medical problems through incorporation of long-tail concepts which are difficult for LLMs to capture effectively^60^. More broadly, many of the observed errors likely reflect the absence of a robust world model—an internal causal representation that enforces physical and logical constraints, maintains latent state, and supports plausibility and counterfactual checks. We observe that this limitation is not unique to medicine and is a central target for improving LLM reasoning in general^61^.

Our findings align with recent evidence that LLMs have limited generalization—the ability to effectively apply reasoning to novel, out-of-distribution scenarios^62,63^. Deficiencies of LLM generalization have been identified in multiple straightforward tasks^18,19,64^, including simple mathematical reasoning^65^, and in planning, even with current state-of-the-art models^62^. Generalization in out-of-distribution contexts is essential in real-world clinical scenarios, which often demand that reasoning strategies used in familiar, predictable situations be countermanded to consider more optimal approaches; this cognitive flexibility is foundational for effective clinical reasoning^66^. LLM inflexibility in reasoning, as demonstrated on mARC-QA, may hinder their ability to generalize to novel or unpredictable scenarios, undermining their reliability in real-world clinical contexts. Subsequently, reduced adaptability to novelty or distribution shifts could undermine healthcare systems’ preparedness for future disease outbreaks—particularly those involving novel pathogens akin to COVID-19.

Compounding these shortcomings are recently demonstrated LLM deficiencies in metacognition—specifically, the inability to recognize their own limitations—and overconfidence^25^. Lack of metacognition and common sense in LLMs can lead to adverse outcomes if they are overrelied upon in clinical contexts^44^. Our findings suggest that LLM limitations in reasoning may be exacerbated in long-tail or out-of-distribution contexts. To mitigate these risks, the development of selective prediction strategies, as proposed by Goetz et al.^55^, may offer a pathway to AI deployment in clinical scenarios. In this strategy, LLMs could defer to human clinicians in long-tail or out-of-distribution scenarios, ensuring that critical decisions are supervised by experts in contexts where LLMs may be unreliable^55^.

We acknowledge several limitations in this study. Compared to prior medical QA benchmarks such as specialty board exams and the USMLE, mARC-QA consists of a smaller set of 100 questions. This reduced number reflects the nontrivial aspect of crafting questions that test long-tail or out-of-distribution reasoning patterns, which are more novel than those found in conventional medical QA. Future work will aim to increase the size of the mARC-QA dataset to improve its robustness. We recognize as well that question curation was based on physician expertise and not systemically performed. There are recently proposed LLM methods to select knowledge that is out-of-distribution (OOD), however, to our knowledge, this is not well-studied in medical contexts. For example, agentic-based OOD question generation has recently been explored in math and science high-school level question contexts, and future iterations of mARC may generate additional questions in this manner^67^. Furthermore, we recognize that expert review of the dataset reflects content validity, not *Einstellung* construct validity, and formal construct validation will be explored in future work.

Additionally, while mARC questions might appear punitive by targeting otherwise beneficial heuristics with “edge-case” scenarios, the intention is to assess “cognitive” flexibility—a key skill in medical practice, where inflexible adherence to heuristics could lead to harm. We recognize that mARC-QA questions are unlikely to be encountered in the real world; however, our aim is not to benchmark “human-like” cognition or predict human competence in real-world clinical reasoning—an aim already addressed by existing assessments like the USMLE and board exams—but to probe failure modes in LLM reasoning in order to target areas where improvement is needed. Whereas conventional QA benchmarks are established at assessing human clinical reasoning, their reliability at evaluating LLM reasoning remains unclear^25^. This limitation highlights the need for complementary stress tests. Accordingly, to improve applicability to real-world practice, future development will explore long-tail, rare diseases to complement our focus on *Einstellung* driven reasoning. Rare disease evaluations probe unusual content^68^, whereas mARC QA probes unusual reasoning. Both types of stress test are needed as they probe distinct failure modes.

Lastly, we observe that human performance on mARC-QA was limited, consistent with long-standing findings that humans may be susceptible to the *Einstellung* effect^69^. The average performance ( 66%)—comparable to typical accuracy on board examinations and in-training assessments^70^—reflects the inherent variability in effort and reasoning abilities among subjects. On the nine items in the human miss-set where the best model (DeepSeek-R1) outperformed humans (Figure 7), qualitatively, inspection of answer explanations (Supplementary Table 7) indicate that the model appeared to properly adhere to constraints in the question stem and follow logical negation, whereas the human majority did not. These observations indicate that reasoning models can occasionally surpass human performance, and the potential of newer reasoning models to overcome *Einstellung* effect will be explored in future work.

mARC-QA reveals limitations in LLM medical reasoning, challenging the notion that human-level performance on medical QA benchmarks suggests robust medical reasoning capabilities. The findings emphasize the need for the development of benchmarks that rigorously assess LLM generalization in medical reasoning, with assessment of reasoning flexibility serving as a potential approach. The observed shortcomings of LLMs in medical reasoning indicate the need for caution when utilizing LLMs in clinical contexts.

## Supplementary Information

Supplementary Information.

## Data Availability

The mARC-QA problem dataset and the code used to generate the results are publicly available at https://github.com/bernardolab/mARC-QA.
