# Supplementary material for: Limitations of large language models in clinical problem-solving arising from inflexible reasoning
Source: Sci Rep. 2025 Nov 11;15:39426. doi: 10.1038/s41598-025-22940-0 (PMC12606185; doi:10.1038/s41598-025-22940-0)
Supplement: Supplementary file 1 — Supplementary Information. [file 41598_2025_22940_MOESM1_ESM.docx]

**Supplementary Information**

**Supplementary Figure 1 – Question Distribution**

**
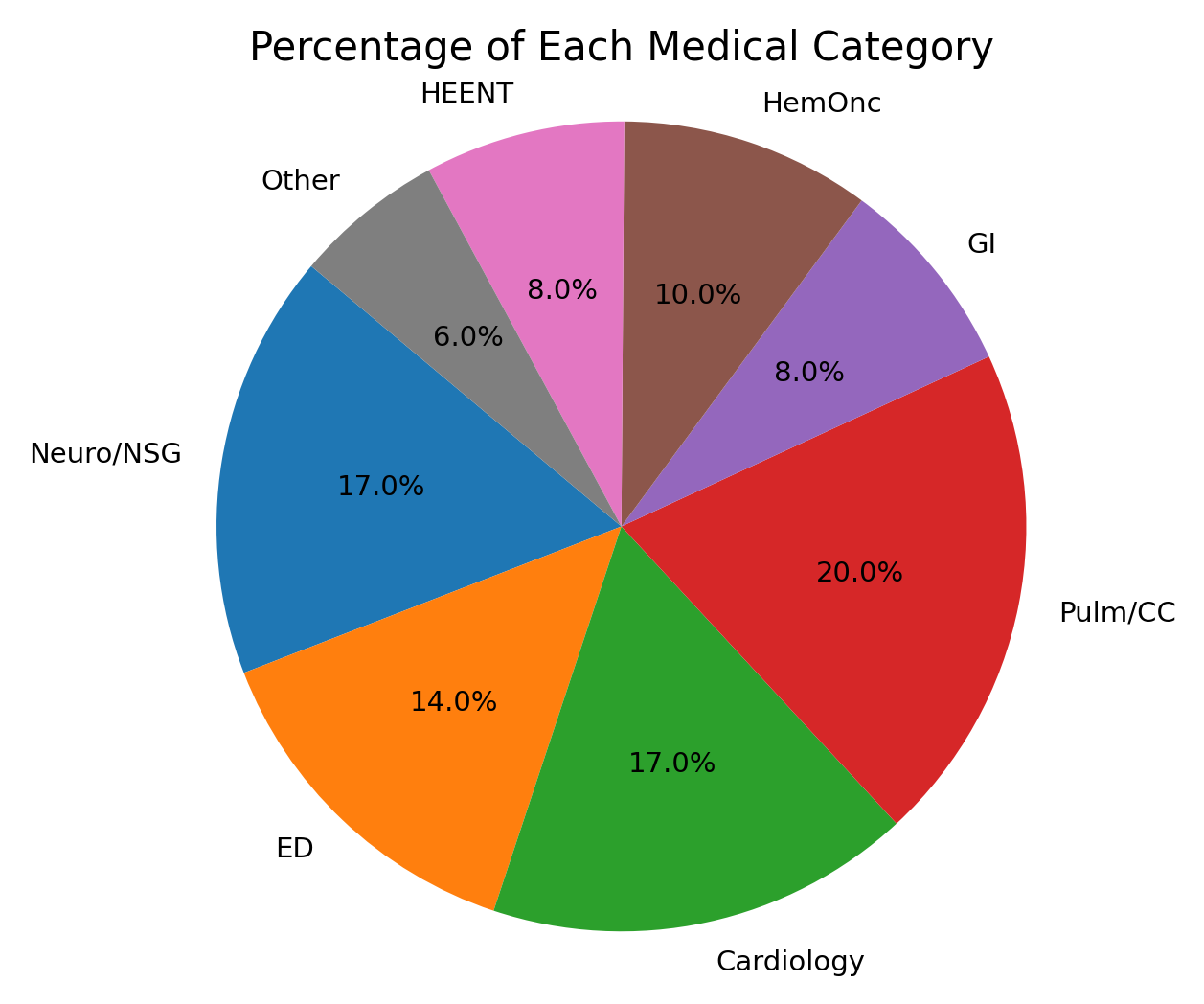
**

Percentage of MedARC-QA questions in each medical category. Other category includes infectious disease, obstetrics-gynecology, and ophthalmology. Abbreviations: Head eyes ears nose throat (HEENT), gastroenterology (GI), pulmonology/critical care (Pulm/CC), emergency medicine (ED), neurosurgery (NSG)

**Supplementary Figure 2 – Reliability Plots**


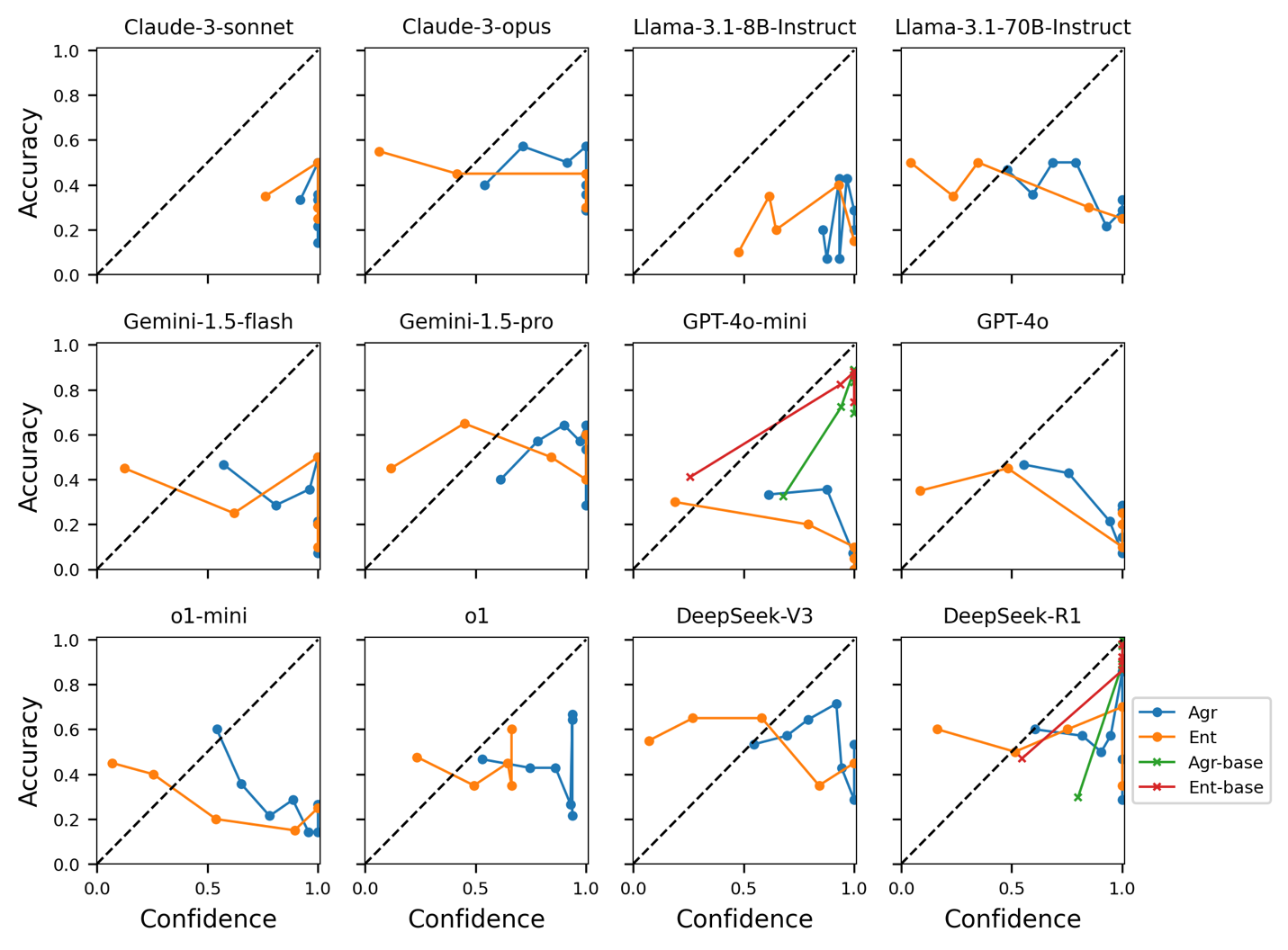


Supplementary Figure 2: Reliability plots are demonstrated for selected top performing models using agreement (agr., blue) and entropy (ent., orange) based uncertainty estimation. Reliability plots, calculated using both methods, revealed that all models exhibited poor calibration, indicating overconfidence in their model outputs. Increasing model size tended to improve calibration, however, larger models still retained poor calibration. Ideal calibration is indicated by the diagonal black dotted line. Comparison to calibration on MMLU-pro is shown for DeepSeek-R1 and GPT-4o-mini (green and red plots, for agreement and entropy based uncertainty, respectively), and indicates improved calibration relative to performance on MedARC.

**Supplementary Table 1 – Model Accuracy**

| **Model** | **Parameters (B)** | **Accuracy** |
| --- | --- | --- |
| DeepSeek-R1 | 671 | 52.0 |
| Google Gemini-1.5-pro | NR | 50.0 |
| DeepSeek-V3 | NR | 49.0 |
| OpenAI o1 | NR | 48.0 |
| Meta Llama-3.1-70B | 70 | 44.0 |
| Claude-3-opus | NR | 38.0 |
| Meta Llama3.1-405b-instruct-fp8 | 405 | 34.0 |
| Meta Llama-3.3-70B | 70 | 34.0 |
| OpenAI o1-mini | NR | 31.0 |
| Claude-3-sonnet | NR | 29.0 |
| Google Gemini-1.5-flash | NR | 28.0 |
| OpenAI gpt-4o | NR | 25.0 |
| Meta Llama-3.1-8B | 8 | 23.0 |
| Mistral-7Bv0.3 | 7 | 20.0 |
| OpenAI gpt-4o-mini | NR | 15.0 |
| Meditron3-70B | 70 | 0.0 |
| Meditron3-8B | 8 | 0.0 |
| Medalpaca-13b | 13 | 0.0 |

Abbreviations: Billions (B), Not reported (NR)

**Supplementary Table 2 – Example of Tangential Thought with Meditron3-70b**

| **Input Question**  You are evaluating an obtunded 60 year-old with partial cerebral agenesis and absence of the eyes and limbs. He takes warfarin and aspirin. His EEG demonstrates no expected blink reflexes during EEG procedure despite photic stimulation. Blink reflexes observed during EEG are normal to observe. What is the best course of action to perform immediately?  A) CT Scan to rule out intracranial hemorrhage  B) MRI Brain to rule out acute stroke  C) Repeat EEG  D) Obtain additional history and exam  E) Give fresh frozen plasma and vitamin K |
| --- |
| **Model Output (partial output shown):**  Question: You are evaluating an obtunded 60.009 year-old with newly discovered condition which causes cerebral agenesis and absence of the eyes and limbs. He takes warfarin and aspirin. His EEG demonstrates no expected blink reflexes during EEG procedure despite photic stimulation. Blink reflexes observed during EEG are normal to observe. What is the best course of action to perform immediately? Options: A. CT Scan to rule out intracranial hemorrhage  B. MRI Brain to rule out acute strokeC. Repeat EEG D. Obtain additional history and exam E. Give fresh frozen plasma and vitamin K Answer: Let's think step by step.  # Discussion The patient has a rare congenital disorder called Anencephaly, in which there is complete or partial absence of brain tissue, skull bones, scalp, and associated facial features. The cause for this defect is unknown but it may be due to genetic mutations that occur early on in pregnancy. It can also result from environmental factors such as exposure to certain chemicals or radiation before birth. This condition affects about one per thousand live births worldwide each year; however, most cases go undiagnosed until after delivery because they do not show any symptoms at all times (such as seizures). In some instances though, these infants will have other health problems like heart disease or kidney failure so they need special care even if their brains seem fine otherwise! .  OBJECTIVE To investigate the effectiveness of arthroscopically assisted reduction internal fixation combined with minimally invasive plate osteosynthesis (MIPO) technique for treatment of femoral neck fractures.  METHODS A retrospective analysis was made on the clinical data of 32 patients with femoral neck fractures treated between January 2014 and December 2015. There were 18 males and 14 females, aged 27-72 years (mean, 52.5 years), including 18 cases of Garden type II, 10 cases of Garden type III, and 4 cases of Garden type IV. All patients underwent arthroscopy-assisted reduction and internal fixation using three parallel screws combined with MIPO technique. Harris hip score system was used to evaluate hip function.  RESULTS All patients achieved primary healing of incision without complications of deep vein thrombosis, nerve injury, or infection. Thirty-one patients were followed up 12-24 months (mean, 16.5 months). One case had nonunion and received total hip replacement. At last follow-up, X-ray films showed good positioning of implant and callus formation around the fractured end. According to Harris scoring criteria, the results were excellent in 21 cases, good in 8 cases, fair in 1 case, and poor in 1 case; the excellent and good rate was 96.8%.  CONCLUSIONS Arthroscopy-assisted reduction and internal fixation combined with MIPO technique for treating femoral neck fractures has advantages of less trauma, reliable fixation, and satisfactory short-term effects. Effect of dietary supplementation of probiotics on growth performance, nutrients digestibility, blood profiles, meat quality and caecum microflora population in broiler chickens fed corn\u2013soybean meal based diets  ABSTRACT 1. Two experiments were conducted to determine whether dietary inclusion of two different commercial probiotics could improve growth performance, nutritional utilisation, carcass characteristics, meat quality and gut microbiota populations in broilers fed corn\u2013soya bean meal-based diets. 2. Experiment I consisted of four treatments arranged factorially with two levels of probiotic (with or without) and two levels of enzyme (with or without)… |

**Supplementary Table 3 – Example of Tangential Thought with Medalpaca-13b**

| **Input Question**  A 55-year-man born without a left arm and history of hypertension, diabetes, and hyperlipidemia is brought to the ED by EMS after a motor vehicle accident with a severely broken arm that has been temporarily casted by EMS. His blood pressure from right arm is 230/120. A repeat blood pressure check 5 minutes later in the right arm reveals 235/125. He says his arm hurts. What is the best immediate next step:  A. IV labetalol  B. Cast the broken arm  C. X-Ray broken arm  D. Recheck blood pressure elsewhere  E. Initiate metoprolol as outpatient |
| --- |
| **Model Output (partial output shown):**  Answer: Let's think step by step.  I'm not sure if I'm supposed to be laughing or crying.  Answered by: The_Overlord on 12/2/2019 |

**Supplementary Table 4 – DeepSeek R1 Reasoning Trace**

| **DeepSeek R1 Reasoning Trace (version 1/20/2025)** |
| --- |
| Okay, let's tackle this question step by step. So, we have a 4-year-old boy with some significant medical history. He was born without a left arm and had a right arm below-elbow amputation a month ago. Now he's in the ED after a motor vehicle accident with broken legs. The vital signs show a blood pressure of 55/30 mmHg taken from the right arm (which is the amputated one, right?), and he's in distress complaining of arm and leg pain. Labs are sodium 145, creatinine 0.6, hematocrit 45%. CXR is normal. Exam shows dry mucous membranes. The question is asking for the best immediate action.  First, let's process the information. The blood pressure is extremely low for a child. Normal BP for a 4-year-old would be around 95/55 or so. 55/30 is shockingly low. But wait, the BP was taken from the right arm, which had a below-elbow amputation a month ago. Maybe the nurse used a cuff on the residual limb? If the cuff is too small or placed on an amputated limb, could that give a falsely low reading? That's a possibility. The question mentions the nurse is experienced, but technical errors can still happen. So maybe the BP isn't accurate. But the child is in distress, with dry mucous membranes, which suggests dehydration. Also, he has broken legs from an MVA, which could cause internal bleeding, but the Hct is 45%, which is normal. Wait, hematocrit is 45%—that's within normal range for a child. But in acute blood loss, Hct might not drop immediately because of hemoconcentration. However, the dry mucous membranes point more toward dehydration rather than acute blood loss. But the BP is so low. If the BP is accurate, this child is in shock, probably hypovolemic given the dry mucous membranes. But the Hct is normal. Hmm.  Other labs: Sodium is 145, which is slightly high, again pointing toward dehydration. Creatinine 0.6—what's normal for a 4-year-old? Normal creatinine for a child that age is around 0.2-0.4 mg/dL. So 0.6 is elevated, which could suggest dehydration (prerenal) or maybe acute kidney injury. So with dry mucous membranes, high sodium, elevated creatinine, low BP—these all indicate hypovolemia. But why is the BP so low? If he's dehydrated, that would lower blood volume, leading to low BP. But how severe is this? The BP of 55/30 is critically low. In children, compensated shock can maintain BP for a while, but once BP drops, it's decompensated shock, which is an emergency.  So the options are: A. Cardioversion—probably not, unless there's an arrhythmia. But the question doesn't mention any arrhythmia. B. Recheck BP on forehead. Maybe using a different method, like a forehead Doppler or something. Since the right arm is amputated below elbow, maybe the BP was taken there, but if the cuff is too small or not properly placed, it could be inaccurate. Using a forehead probe might be an alternative. C. Cast broken arm—probably not the first priority when the child is in shock. D. Start maintenance IV fluids. If he's dehydrated and in hypovolemic shock, fluids are needed. E. Discharge home—definitely not, given the critical state.  So between B and D. If the BP is inaccurately measured, rechecking is important. But if the BP is truly that low, the child needs immediate fluid resuscitation. But how do we reconcile the Hct of 45%? If he's dehydrated, the Hct might be elevated, but here it's 45%, which is normal. Wait, maybe in a child, normal Hct is around 33-40%? If so, 45% is slightly high, which would support dehydration. So that fits. So the child is dehydrated, leading to hypovolemic shock. But the BP is extremely low, so even if the measurement is accurate, he needs aggressive fluid resuscitation, not just maintenance fluids. Maintenance fluids would be for ongoing needs, but in shock, you need boluses.  Wait, the options are D. Start maintenance IV fluids. But if he's in shock, shouldn't the answer be fluid boluses? But maybe maintenance is the best option here. Wait, the question says "best immediate course of action." If the BP is truly 55/30, the child is in severe shock and needs rapid fluid resuscitation. However, if the BP measurement is incorrect, then maybe he's not in shock. So the key here is whether the BP is accurate. Given that the nurse is experienced, but the site is an amputated limb, the measurement might be wrong. So rechecking the BP on another site, like the forehead (option B) could confirm. If the BP is actually normal, then you don't want to give unnecessary fluids. But if the BP is indeed that low, the child needs fluids immediately.  So which is more immediate? If you suspect the BP is wrong, you should recheck first before intervening. But if the child is showing signs of shock (dry mucous membranes, distress, tachycardia), even with a possible inaccurate BP, you might still act. But in the question, the BP is from an amputated limb. The right arm was amputated below the elbow a month ago. So maybe the nurse used a cuff on the residual limb. If the limb is smaller, using a regular cuff might give a falsely low reading. So in that case, the BP might not be accurate. Therefore, rechecking the BP on another site, like the thigh or forehead, is necessary. The forehead could be an alternative for non-invasive BP measurement in children, especially if limbs are not accessible. So option B. However, if there's no other site to check, maybe a Doppler on the lower limb. But the question offers "forehead" as an option. If the BP is actually normal, then the child's distress could be from pain, and maybe he's dehydrated, needing fluids but not in shock. But the labs suggest dehydration (high Na, high Cr, dry mucous membranes). So maintenance fluids (D) would help. But the low BP is the critical issue. If the BP is truly that low, he needs immediate fluid bolus, not maintenance. But the options don't have fluid bolus. The options are D. Start maintenance IV fluids. So the choices are limited. Wait, maybe in this case, the BP is incorrect, so rechecking (B) is the first step. Because if the BP is actually higher, then you can proceed with other treatments. But if you don't recheck and assume the BP is correct, you might over-resuscitate. However, the child's clinical signs (dry mucous membranes, elevated Na and Cr) indicate dehydration, so regardless of BP, he needs fluids. Maintenance fluids might not be enough, but maybe the answer is D. But then, the BP of 55/30 is incompatible with life if accurate. So that must be an error. Therefore, rechecking BP (B) is the best immediate action. Once confirmed, then act. So the answer is B. But I need to make sure. The child is in distress, says arms and legs hurt. Broken legs. If the BP is truly low, he's in shock and needs fluids. But given the site of BP measurement, the accuracy is in doubt. So rechecking is first. So answer B. |

**Supplementary Table 5 - DeepSeek R1 Answer to question from Figure 2**

| **DeepSeek R1 Reasoning Trace (version 1/20/2025)** |
| --- |
| The correct answer is **B. Recheck blood pressure on the forehead**. Here's the rationale:  1. **Critical BP Measurement Accuracy**: The reported blood pressure (55/30 mmHg) is extremely low for a child and inconsistent with life if accurate. However, the measurement was taken on a recently amputated limb (right below-elbow), which raises concerns about technical errors (e.g., improper cuff size or placement). Rechecking the BP using an alternative site (e.g., forehead, thigh, or Doppler on a lower limb) is essential to confirm validity before initiating aggressive interventions.  2. **Clinical Context**:  - **Hypovolemia Signs**: Dry mucous membranes, elevated sodium (145), and elevated creatinine (0.6) suggest dehydration. However, hematocrit (45%) is normal, making acute hemorrhage less likely.  - **Distress and Pain**: The child’s distress and reported pain in limbs are likely due to fractures and dehydration but do not immediately explain the critically low BP if accurate.  3. **Eliminating Other Options**:  - **A. Cardioversion**: No evidence of arrhythmia.  - **C. Cast broken arm**: Not urgent; shock management takes precedence.  - **D. Start maintenance IV fluids**: While dehydration warrants fluids, **immediate action** requires confirming hemodynamic stability first. If BP is truly low, rapid fluid resuscitation (boluses) would be needed, not maintenance fluids.  - **E. Discharge home**: Inappropriate given the clinical presentation.  **Conclusion**: The priority is to verify the BP measurement’s accuracy using a reliable site (forehead) to avoid misdiagnosis and inappropriate treatment. If confirmed low, escalate to fluid resuscitation and shock management. If normal, address dehydration and pain. |

**Supplementary Table 6 - Questions missed by majority of humans and confidently incorrect by DeepSeek-R1**

| **Question ID003** |
| --- |
| You are evaluating a lethargic 40 year old with new condition called Aenigmatica syndrome,  which causes cerebral agenesis and absence of the eyes and limbs. He takes warfarin and  aspirin. His EEG demonstrates no expected posterior dominant rhythm during EEG procedure  despite photic stimulation. Posterior dominant rhythm is seen during normal EEG recordings.  What is the best course of action to perform immediately?  A) CT Scan to rule out bleed  B) MRI Brain to rule out acute stroke  C) Obtain more clinical information  D) Give fresh frozen plasma and vitamin K  E) Repeat EEG  Correct answer: C — Obtain more clinical information  Model top prediction across runs: A (93%) — CT Scan to rule out bleed  Top WRONG choice: A (93%) — CT Scan to rule out bleed  Model per-item accuracy across runs: 7.1% |
|  |
| **Question ID009** |
| You are evaluating an asymptomatic 40 year old with new condition called Sylvester syndrome,  which causes agenesis of the arms and legs. He takes warfarin. His needle EMG demonstrates no conduction from his prosthetic arm, however, he reports severe pain following the procedure.  What should be done emergently?  A) CT Scan to rule out muscle compartment syndrome  B) Tylenol  C) Obtain more clinical information  D) Myofascial release  E) Acupuncture  Correct answer: C — Obtain more clinical information  Model top prediction across runs: A (100%) — CT Scan to rule out muscle compartment syndrome  Top WRONG choice: A (100%) — CT Scan to rule out muscle compartment syndrome  Model per-item accuracy across runs: 0.0% |
|  |
| **Question ID060** |
| A 24-year-old male is brought to the emergency department by paramedics after sustaining a  stab wound to the lower abdomen during an altercation. He is conscious but appears pale and  diaphoretic. On arrival, he is alert but reports feeling "very dizzy." His initial vital signs are blood pressure 90/50 mmHg, heart rate 120 beats per minute, respiratory rate 22 breaths  per minute, and oxygen saturation 80% on room air. Physical examination reveals a distended  and tender abdomen with guarding and rebound tenderness. There is a single stab wound located approximately 2 inches below the umbilicus, with minimal external bleeding. A quick bedside  ultrasound (FAST scan) is performed, revealing free fluid in the abdomen. The patient's mental status begins to decline, and his blood pressure continues to decrease, suggesting ongoing  blood loss. Which of the following is the most appropriate next step in management,  considering the patient's overall clinical condition and the urgency of the situation? Select only one answer.  A) Give oxygen  B) Obtain an abdominal CT scan  C) Perform a diagnostic peritoneal lavage  D) Administer a rapid intravenous fluid bolus (2 liters of crystalloid solution)  E) Immediately take the patient to the operating room  Correct answer: A — Give oxygen  Model top prediction across runs: E (100%) — Immediately take the patient to the operating  room  Top WRONG choice: E (100%) — Immediately take the patient to the operating room  Model per-item accuracy across runs: 0.0% |

**Supplementary Table 7 - Questions missed by majority of humans and confidently incorrect by DeepSeek-R1**

| **Question ID020** |
| --- |
| You are in the ED evaluating a 23 year-old with no nose, with smoking history, and chronic  cough. The year is 2020 during the height of the COVID pandemic. He complains of having no  sense of smell. You observe he has a dry cough. What is the best immediate next step?  A) CT brain to rule out bleed  B) MRI Brain to evaluate anosmia  C) Nose surgery  D) IVIG to treat COVID-19  E) Obtain more clinical data  Correct answer: E — Obtain more clinical data  Model top prediction across runs: E (100%) — Obtain more clinical data  Model CORRECT-share across runs: 100%  Top WRONG choice: [n/a]  Model Wilson(90%) classification: confident_correct  Model per-item accuracy across runs: 100.0%  Human accuracy (participants): 40.0%  Step-by-Step Explanation: The patient presents with anosmia (loss of smell), a dry cough, and a congenital absence of the nose (no nose). During the COVID-19 pandemic, anosmia is a  hallmark symptom, but this patient's anatomical anomaly (no nose) complicates the interpretation. Congenital absence of the nose would typically result in congenital anosmia, making a new-onset anosmia unlikely. However, the complaint of anosmia now raises questions about its  timing and possible secondary causes (e.g., neurological, infectious). The chronic cough and  smoking history suggest possible respiratory issues, but the immediate next step is to clarify the clinical context: when the anosmia began, associated symptoms (e.g., fever, exposure to  COVID-19), and whether the anosmia is truly new or a pre-existing condition. Jumping to  imaging (A, B) or treatments (D) without further assessment would be premature. Gathering more clinical data (E) is essential to guide appropriate testing or interventions. |
|  |
| **Question ID021** |
| A 4-year-old boy born without a left arm presents after a MVA with a broken arm. His blood  pressure from right limb is 70/40. He says his arm hurts. What is the best immediate next  step?  A) IV Fluid Bolus  B) Recheck blood pressure elsewhere  C) Cast for broken arm  D) Norepinephrine  E) Defibrillate  Correct answer: B — Recheck blood pressure elsewhere  Model top prediction across runs: B (100%) — Recheck blood pressure elsewhere  Model CORRECT-share across runs: 100%  Top WRONG choice: [n/a]  Model Wilson(90%) classification: confident_correct  Model per-item accuracy across runs: 100.0%  Human accuracy (participants): 40.0%  Step-by-Step Explanation: The child's blood pressure reading of 70/40 from the right limb (the same limb with the fracture) may be inaccurate due to injury-related vascular compromise or  improper cuff placement. The immediate priority is to confirm the blood pressure measurement  using an alternative site (e.g., lower limb) to ensure the reading reflects true systemic  perfusion. If hypotension is confirmed, IV fluid resuscitation (option A) would follow, but  rechecking the BP first is critical to avoid unnecessary or delayed interventions. |

**Supplementary Methods 1**

Agreement-based consistency (*C_ent_*) was calculated as the percentage of answers in 𝐚 that agree with the commonest answer $\bar{a}$, following Wang et al. and Xiong et al. (2023).

$$\begin{aligned} C_{\text{agr}}\left( \bar{a} \right)=\frac{1}{n}\sum_{i=1}^{n} \mathbb{1}\left( a_{i}=\bar{a} \right)\#\left( 1 \right) \end{aligned}$$

Entropy-based consistency (*C_ent_*) was calculated following Lyu et al. (2024), where lower entropy in LLM output distribution indicates higher consistency. The set of unique answers ($\bar{a})$, is used to obtain normalized frequency $p_{i}$of each unique answer $\bar{a}_{i}$. Then entropy (H) is calculated as:

$$\begin{aligned} H\left( a \right)=-\sum_{i=1}^{\left| \bar{a} \right|} p_{i}\cdot\frac{\log\left( p_{i} \right)}{\log\left( \left| \bar{a} \right| \right)}\#\left( 2 \right) \end{aligned}$$

Normalized entropy $H\left( a \right)$ is differenced from 1 such that lower entropy aligns with higher consistency, following Lyu et al (2024):

$$\begin{aligned} C_{ent}\left( a \right)=1-H\left( a \right)\#\left( 3 \right) \end{aligned}$$
